# Acute dacryocystitis with giant lacrimal abscess: a case report

**DOI:** 10.1186/s13052-020-0779-7

**Published:** 2020-02-03

**Authors:** Maria Di Cicco, Elisabetta Maria Bellino, Andrea Marabotti, Laura Luti, Diego G. Peroni, Giampiero I. Baroncelli

**Affiliations:** 1 Pediatrics Unit, Pisa University Hospital, Via Roma n. 67, 56126 Pisa, Italy; 2 Ophthalmic Surgery Department, Pisa University Hospital, Pisa, Italy

**Keywords:** Pediatric acute dacryocystitis, Nasolacrimal duct obstruction, Lacrimal abscess, Dacryocystorhinostomy, Dacryocystectomy

## Abstract

**Background:**

We report a case of a 4-year-old girl with acute dacryocystitis complicated with giant lacrimal abscess who underwent open dacryocystectomy as resolutive surgery.

**Case presentation:**

A 4-year-old previously healthy girl presented to the emergency department with a voluminous and erythematous, fluctuant warm mass localized inferiorly to the medial canthus of the right eye. She had a 2-week history of right inferior eyelid oedema and hyperemia, treated firstly with dexamethasone and netilmicin by eye drops, and then with per oral amoxicillin clavulanate. Ultrasound examination showed a well-circumscribed round lesion filled by anechoic fluid with punctate echoes, confirming a diagnosis of acute dacryocystitis complicated by lacrimal abscess. Parents refused a head CT. Systemic antibiotic treatment was started and, on 5th day from admission, open dacryocystectomy was performed with good esthetical result.

**Conclusions:**

Pediatric acute dacryocystitis is a potentially serious condition, which must be treated with intravenous antibiotic therapy followed by surgery tailored to the clinical history. Even if probing and dacryocystorhinostomy are the most used surgery in adults and children, open dacryocystectomy is a safe and successful option, mainly in severe cases where imaging studies are not available.

## Background

Pediatric Acute Dacryocystitis (PAD) is a quite rare and potentially serious condition, characterized by acute inflammation of the lacrimal sac, which can rapidly evolve into lacrimal sac abscess or other serious life-threatening complications [1, 2]. PAD is mostly caused by congenital nasolacrimal duct (NLD) obstruction: therefore, it is not uncommon in newborns and infants but is quite rare in older children [3]. We report a case of PAD complicated with giant lacrimal abscess in a 4-year-old previously healthy girl. Even if systemic antibiotic treatment combined with probing and dacryocystorhinostomy (DCR) is the most used approach to treat acute dacryocystitis in adults and children, in our patient open dacryocystectomy (DCT) was performed, demonstrating that this could be considered a safe and successful option in selected patients.

## Case presentation

**Fig. 1:**
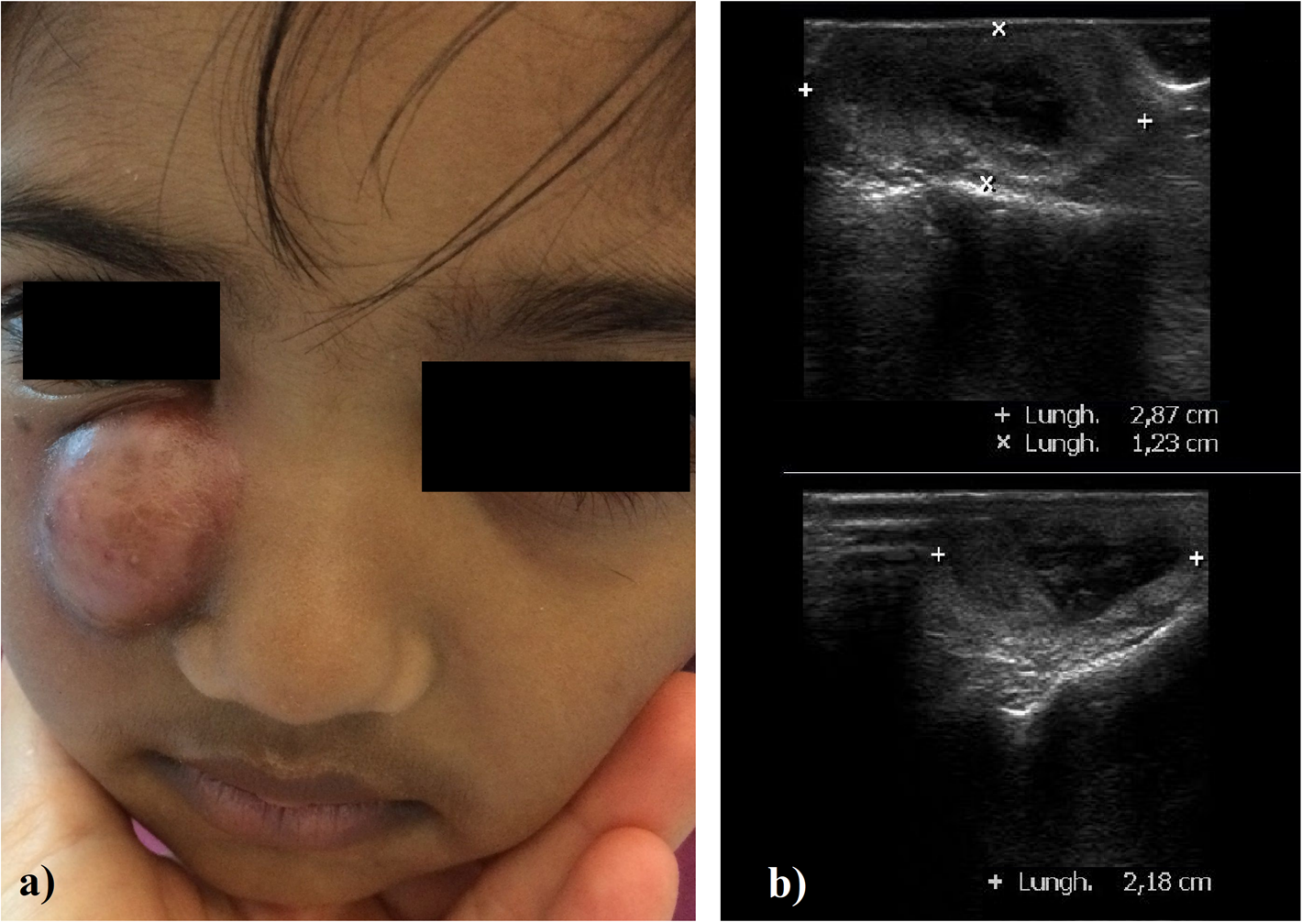
**a** Physical examaination at day 1: our patient had a voluminous and erythematous mass localized inferiorly to the medial canthus of the right eye, without purulent drainage or signs of conjunctivitis. **b** Ultrasound diagnostic aspects of the mass: a well-circumscribed round lesion filled by anechoic fluid is clearly detectable

**Fig. 2:**
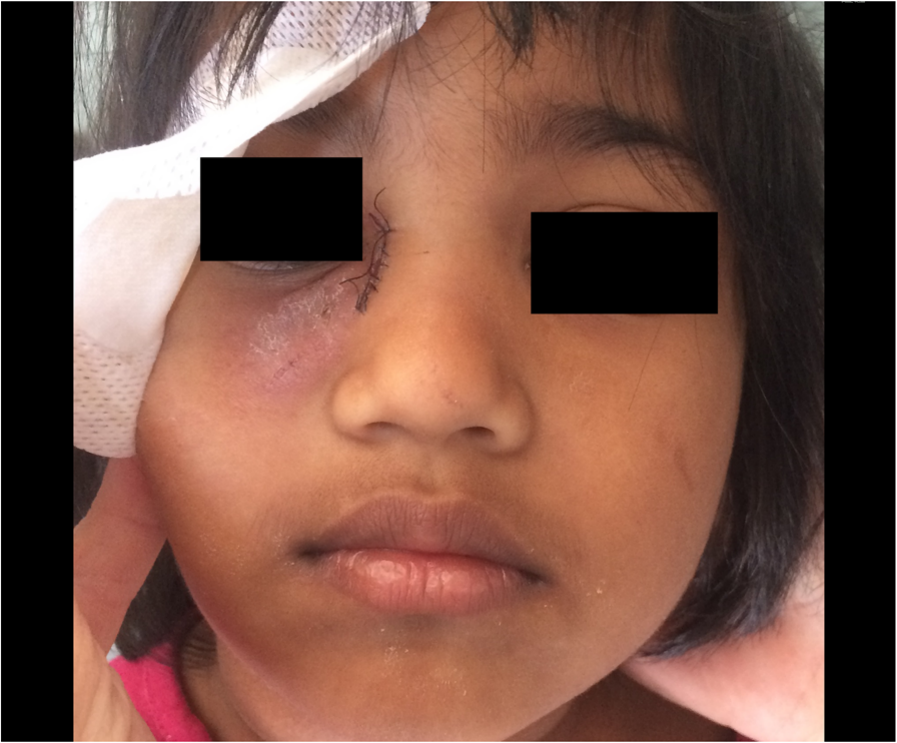
Physical examaination at day 6, after open dacryocystectomy was performed

A 4-year-old Bengali girl with no significant past medical history was brought to our emergency department with a 2-week history of right inferior eyelid oedema and hyperemia, treated firstly with dexamethasone and netilmicin by eye drops, and then with per oral amoxicillin clavulanate. Despite this treatment her symptoms rapidly worsened: on evaluation she had a voluminous and erythematous, fluctuant warm mass localized inferiorly to the medial canthus of the right eye, without purulent drainage by applying pressure over the lacrimal sac area or signs of conjunctivitis (Fig. 1a). She was in good general conditions and she had neither fever nor impairment in visual and ocular motility. No history of previous signs of NLD obstruction or facial trauma was reported. Ultrasound examination showed a well-circumscribed round lesion (dimensions 2,9 cm × 1,2 cm × 2,2 cm) filled by anechoic fluid with punctate echoes, confirming the diagnosis of PAD complicated by lacrimal abscess (Fig. 1b). White-cell count was 7810 per cubic millimeter (reference range, 7500 to 15,500); C-reactive protein and procalcitonin were in the normal range. Parents refused a head CT, so that we could not investigate the presence of conditions causing NLD obstruction. She was immediately treated with intravenous teicoplanin (20 mg/kg in the first day, followed by 10 mg/kg/day), and piperacillin/tazobactam (100 mg/kg/day). However, 3 days later the lesion showed a rapid and progressive colliquation with skin fistula and leakage of abundant purulent material. Culture of the drainage fluid grew methicillin-sensitive *Staphylococcus aureus*. On 5th day from admission, open DCT was performed with good esthetical result (Fig. 2). Anatomopathological examination of surgical specimen ruled out tumors and malformations. She was discharged on postoperative day 6 on a 7-day regimen of per oral clindamicin (30 mg/kg/day). Follow up 2 weeks and 2 months after surgery revealed no swelling recurrence.

## Discussion and conclusions

PAD is a potentially serious condition characterized by acute inflammation of the lacrimal sac, defined as “*a medical urgency which is clinically characterized by rapid onset of pain, erythema and swelling, classically below the medial canthal tendon with or without preexisting epiphora mainly resulting from the acute infection of the lacrimal sac and perisac tissues*” [1]. This is a quite rare condition in children, with the exception of newborns: 6% of healthy newborns have congenital NLD obstruction and, among them, only 2.9% develop PAD. Clinical diagnosis is based on the typical signs and symptoms and this condition is usually unilateral and more common in females [1, 2]. In a recent case series (320 patients), swelling in the lacrimal sac area with acute onset pain were the most common presenting symptoms (84.4%), while lacrimal abscess was the presenting feature in 23% of the patients [1]. Epiphora and fever are variably associated with infection [1, 3].

Compared to adult dacryocystitis, PAD shows a much more rapid progression associated with pain, redness and swelling of the skin over the medial canthal region up to a lacrimal abscess, as in our case. Progressive orbital cellulitis, superior ophthalmic vein thrombosis, cavernous sinus thrombosis, and septicaemia may occur in untreated patients with PAD. Complete vision loss has been reported, too [4]. Children are at particular risk for complications due to their immature immune system and narrow confines of the drainage system [5].

Formation of a fistula between the lacrimal sac and the skin is a rare event. In the cited case series, 5.6% of patients developed a fistula (83.3% following a spontaneous rupture of the lacrimal abscess and 16.7% secondary to incision and drainage) [1].

PAD is mainly caused by distal congenital NLD obstruction which causes stagnation of tears and debris in the lacrimal sac, promoting bacterial growth and infection [5]. However, acquired etiologies such as sinusitis, facial traumas, foreign bodies, and direct microbial inoculation have been reported [3].

Bacterial cultures from the purulent discharge (sampled at the sac, fistula or abscess surgical incision) are commonly performed [3]; both in children and in adults, the most frequently isolated germ is methicillin-sensitive *Staphylococcus aureus*, followed by *Streptococcus pneumoniae*. Gram negative species can be detected in culture positive acute dacryocystitis too [1, 6]*.*

Differential diagnosis includes some rare conditions as hemangiomas, nasal gliomas, encephaloceles, dermoid and epidermoid cysts and lymphangiomas [5].

Imaging studies (ultrasonography, CT or MRI) are not generally advised but they could be performed in the suspicion of post-traumatic PAD and sinusitis or in case of severe and rapidly progressive course, proptosis or globe displacement [3, 5]. Management of PAD includes antibiotics combined with surgery. PAD can be classified into 4 categories: 1) acute dacryocystitis in neonates requiring systemic antibiotics and probing with or without simultaneous marsupialization of intranasal cysts; 2) acute dacryocystitis with periorbital cellulitis requiring intravenous antibiotics and probing; 3) post-traumatic acute dacryocystitis requiring systemic antibiotics and DCR with stents; 4) acute dacryocystitis complicated by orbital abscess, requiring systemic antibiotics, orbital abscess drainage with simultaneous probing and placement of stents [2, 3]. Usually, treatment of PAD includes penicillins, cephalosporins, clindamycin or vancomycin, but there is no consensus about therapy duration [3]. Complete resolution of PAD with conservative therapy alone has been reported in 23% of patients [5]. Therefore, intravenous antibiotic therapy is usually followed within 1–2 days by surgery tailored to the clinical history [2]. Considering that PAD is mainly caused by NLD obstruction, probing is the surgical intervention of choice once infection is resolved, in order to avoid recurrence [3, 7]. If a lacrimal abscess develops, incision and drainage may be needed. External or endoscopic DCR provides the definitive treatment in adults but this surgery approach is used in children only if recurrent attacks occur or in patients refractory to probing, considering that it is hampered by narrow anatomical spaces and need for special instrumentation. In our patient the ophtalmologists suggested DCT removing the lacrymal sac. DCT is usually performed in childhood when young patients present lacrymal sac mucopyocele with fistula (a), when it is not possible to exclude lacrymal sac tumors (b), in traumas (c), in patients with co-morbidities (d), in multiple times failed DCR (e) or when it is not possible to exclude nasal malformations/hypoplasias (f) [8]. In our patient we were facing a), b) and f) conditions, as she had a large lacrymal sac mucopyocele with cutaneous fistula and CT examination could not be performed in order to exclude tumors and nasal malformations or hypoplasias.

In conclusion, PAD is a potentially serious condition, which must be treated rapidly with intravenous antibiotic therapy followed by surgery tailored to the clinical history. Even if probing and DCR are the most used surgery in adults and children, open DCT is a safe and successful option, mainly in severe cases where imaging studies are not available.

## Data Availability

Data sharing is not applicable to this article as no datasets were generated or analysed during the current study.

## Abbreviations

CT: Computed Tomography
MRI: Magnetic Resonance Imaging
NLD: Nasolacrimal duct
PAD: Pediatric Acute Dacryocystitis
